# Necrology

**Published:** 1895-09

**Authors:** 


### Necrology.

At the meeting of the Mississippi Valley Dental Society, April 18th, 1895, the following action was had.

Drs. Harlan and Betty, Committee on Necrology, reported as follows:

Resolutions on the death of Dr. J. J. R. Patrick, of Belleville, Illinois.

WHEREAS: It has pleased an all-wise Providence to remove from our midst John J. R. Patrick, D.D.S., long a member of this organization, and a conspicuous figure in many dental societies in this country, as well as a prominent citizen of Illinois:

*Therefore, Be it resolved,* that in the death of Dr. Patrick dental surgery and dental science has lost one of its most ardent devotees and distinguished members; and this Society desires to express its sorrow at the loss of one of its fellows who had so long and honorably maintained the dignity of the profession.

*Resolved,* That a copy of these resolutions be forwarded to the family of the deceased, and that they be spread on the records of the Society.

A. W. HARLAN,
E. G. BETTY,
*Committee.*

On motion the report was received and adopted, and ordered spread upon the minutes. As a mark of respect the vote taken was by all members rising in their seats.

